# Assessment of vegetation growth and drought conditions using satellite-based vegetation health indices in Jing-Jin-Ji region of China

**DOI:** 10.1038/s41598-021-93328-z

**Published:** 2021-07-02

**Authors:** Rengui Jiang, Jichao Liang, Yong Zhao, Hao Wang, Jiancang Xie, Xixi Lu, Fawen Li

**Affiliations:** 1 State Key Laboratory of Eco-Hydraulics in Northwest Arid Region of China, Xi’an University of Technology, Xi’an, 710048 China; 2 State Key Laboratory of Simulation and Regulation of Water Cycle in River Basin, China Institute of Water Resources and Hydropower Research, Beijing, 100038 China; 3 Department of Geography, National University of Singapore, Kent Ridge, 117570 Singapore; 4 State Key Laboratory of Hydraulic Engineering Simulation and Safety, Tianjin University, Tianjin, 300072 China

**Keywords:** Climate sciences, Hydrology

## Abstract

Terrestrial vegetation growth activity plays pivotal roles on regional development, which has attracted wide attention especially in water resources shortage areas. The paper investigated the spatiotemporal change characteristics of vegetation growth activity using satellite-based Vegetation Health Indices (VHIs) including smoothed Normalized Difference Vegetation Index (SMN), smoothed Brightness Temperature (SMT), Vegetation Condition Index (VCI), Temperature Condition Index (TCI) and VHI, based on 7-day composite temporal resolution and 16 km spatial resolution gridded data, and also estimated the drought conditions for the period of 1982–2016 in Jing-Jin-Ji region of China. The Niño 3.4 was used as a substitution of El Niño Southern Oscillation (ENSO) to reveal vegetation sensitivity to ENSO using correlation and wavelet analysis. Results indicated that monthly SMN has increased throughout the year especially during growing season, starts at approximate April and ends at about October. The correlation analysis between SMN and SMT, SMN and precipitation indicated that the vegetation growth was affected by joint effects of temperature and precipitation. The VCI during growing season was positive trends dominated and vice versa for TCI. The relationships between VHIs and drought make it possible to identify and quantify drought intensity, duration and affected area using different ranges of VHIs. Generally, the intensity and affected area of drought had mainly decreased, but the trends varied for different drought intensities, regions and time periods. Large-scale global climate anomalies such as Niño 3.4 exerted obvious impacts on the VHIs. The Niño 3.4 was mainly negatively correlated to VCI and positively correlated to TCI, and the spatial distributions of areas with positive (negative) correlation coefficients were mainly opposite. The linear relationships between Niño 3.4 and VHIs were in accordance with results of nonlinear relationships revealed using wavelet analysis. The results are of great importance to assess the vegetation growth activity, to monitor and quantify drought using satellite-based VHIs in Jing-Jin-Ji region.

## Introduction

The Fifth Assessment Report (AR5) of the Intergovernmental Panel on Climate Change (IPCC) showed that the global average surface air temperature had increased by 0.85 ± 0.21 °C from 1880 to 2012. The special report of global warming of 1.5 °C revealed that human activities were expected to have caused approximately 1.0 °C of global warming with respect to pre-industrial levels. Global warming is likely to reach 1.5 °C between 2030 and 2052 if the warming trend continues. Temperature has risen faster at higher northern latitudes, where average temperature has raised nearly twice the rate of global average over past 100 years. More extreme weather and climate events have been observed since 1950s, some of which have been linked to human influences, including increasing trends of extreme high temperature and heavy precipitation events in a number of regions^1,2^, especially, in the twenty-first century, extreme climate events have been occurring more widespread, frequently and in great severity globally^3,4^. A warmer world is expected to have higher temperature variability, which will result in more drought events^5^. For example, China has experienced several severe drought events for past several decades, which profoundly affected the water resources and vegetation growth, and consequently brought greater challenges to agricultural development and water-energy-food security^6,7^. Drought has destructive impacts on crop yields in China, especially in plain regions with thriving agricultural development, which has caused great economic losses^8,9^.

Drought is a recurring phenomenon occurred naturally with significant impacts on human, agricultural and environmental activities across the world^10^. It is one of the most severe natural disasters that exert devastating influences on regional economic, agricultural, ecological and environmental conditions^11^. The severity and frequency of drought are expected to increase in the future, mainly results from decreasing trends of regional precipitation, increasing trends of temperature and evaporation driven by global warming^12^. Generally, drought originates from precipitation deficiency for an extended period and the influences of drought accumulated slowly in most cases. However, it is difficult to determine the onset, end and severity of the drought^13,14^. Various drought indices such as Palmer Drought Severity Index (PDSI), Crop Moisture Index (CMI), Drought Area Index (DAI), Normalized Soil Water Index (NSWI), Reconnaissance Drought Index (RDI), self-calibrating PDSI (sc_PDSI), Soil Moisture Anomaly Percentage Index (SMAPI), Standardized Precipitation Index (SPI), Standardized Precipitation Evapotranspiration Index (SPEI) have been proposed to monitor, quantify and analyze the drought events and their characteristics mainly from three aspects including intensity, duration and spatial coverage^15–17^. These indices were usually used to measure the departure from the normal condition in a moisture variable based on historical distribution^18–19^. However, different drought indices probably lead to some differences both in values and characteristics^20^. Especially, it is difficult to quantify the moisture, thermal and drought conditions merely based on station-based meteorological or hydrological indices.

To avoid the weakness of conventional systems and drought indices to monitor drought, a new type of satellite-based drought index was proposed to describe the cumulative moisture, temperature and vegetation health conditions, including Vegetation Condition Index (VCI), Temperature Condition Index (TCI) and Vegetation Health Index (VHI), which have been widely used to monitor and detect drought-related vegetation conditions. The satellite-based Vegetation Health Indices (VHIs) have been used to characterize drought and vegetation productivity in many previous studies around the world^5,21–26^. For example, Kogan et al.^5^ studied the trends of global and regional drought area for several drought intensities based on the satellite-based vegetation health indices during the warmest decade, especially, two recent strongest droughts happened in Russia and USA in 2010 and 2011, respectively. Li et al.^24^ and Pei et al.^25^ investigated the changing characteristics of drought and also detected the vegetation responses to weather-related variations using VHIs, which provided references for vegetation growth activity monitoring and water resources management. The VHIs has been used as one of useful indices to detect drought characteristics, to quantify the impacts of drought, to monitor regional vegetation growth and assess agriculture production^26^. Besides, the VHIs were further used to estimate the extreme wildfires^17^, crop yield^27^, malaria cases^28^, and design insurance contracts for drought-related yield losses^29^.

To reveal the change mechanism of vegetation growth activity, the paper further studied the linear and nonlinear relationships between VHIs and El Niño Southern Oscillation (ENSO), which is one of the coupled atmosphere–ocean global climate anomalies. ENSO is a recurring global climate oscillation involving changes of water temperature in the central and eastern tropical Pacific Ocean. A warming of the ocean surface or above average sea surface temperature (SST) indicates El Niño event, and in reverse for La Niña event^30–31^. It has been verified that ENSO had noticeable impacts on the vegetation growth in many regions around the world through global teleconnections. Many previous studies have investigated the relationships between vegetation growth and ENSO at different spatial scales and time periods. For example, Zhao et al.^32^ found that the ENSO is the leading climatic driver of interannual variability of Normalized Difference Vegetation Index (NDVI) during growing season to local and remote climate oscillations, especially in some regions such as southern and eastern African, northeastern Asia and northern South America for the period of 1982–2013. Asoka and Mishra^33^ developed a model to predict vegetation health using Niño 3.4 as predictor at one to three months lead time in India, which provides references for early warning and better planning in water resources and agricultural management. Erasmi et al.^34^ detected a close relation of ENSO warm events and periods of reduced vegetation greenness with a 12-month lag using annual NDVI during 1982–2010. The relationships between vegetation growth and ENSO provide the possibility to investigate the impacts of ENSO on vegetation growth using VHIs.

The superiority of satellite-based VHIs relative to other drought indices makes it one of the widely used indices to monitor drought and to assess vegetation growth activity, especially in large area with limited water resources. The Jing-Jin-Ji region includes Beijing city, Tianjin city and Hebei province which has thirteen cities including eleven prefecture-level cities and two county-level city, and it is the biggest urbanized region in North China Plain and one of the three largest regional economic communities in China. The rapid urbanization and economic development prompted the water demands to increase dramatically. Previous studies found that the Jing-Jin-Ji region had a dry tendency mainly located in the northern part for past several decades, which mainly affected the crop yield during growing season^35–36^. However, it is difficult to comprehensively assess the vegetation productivity together with drought characteristics using merely meteorological or hydrological monitoring data. The satellite-based VHIs provide advantages to assess the vegetation growth activity and analyze the drought characteristics. Therefore, the analysis of satellite-based VHIs is an important indicator to assess vegetation growth activity and help to monitor drought, which should be useful for the disaster prevention and reduction decision in Jing-Jin-Ji region.

The primary objectives of this study are: (1) to assess the spatiotemporal variations of vegetation growth activity using long duration (1982–2016) satellite-based vegetation health indices in Jing-Jin-Ji region; (2) to estimate the drought severity and vegetation condition using different categories of VHIs; (3) to reveal the vegetation sensitivity to ENSO based on VHIs using correlation and wavelet analysis. The paper is organized as follows: Material and methods are described in section "Material and methods", followed by results in section "Results" and discussion in section "Discussion and conclusions", and conclusions are presented in section "Definition of satellite-based VHIs".

## Material and methods

### Study area

**Figure 1:**
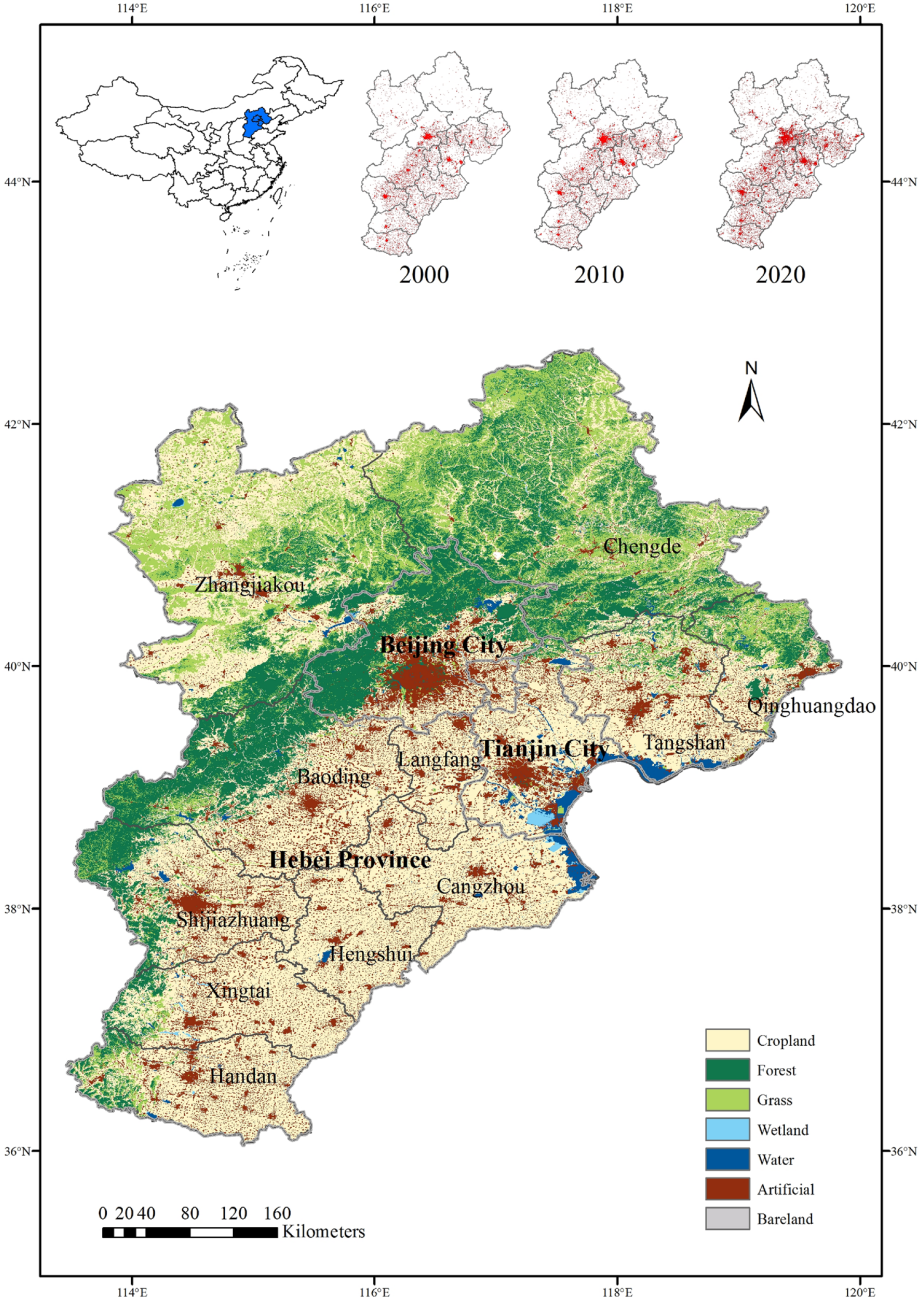
Location and land cover of the study area. The land cover map was extracted from Globeland30 dataset provided by National Geomatics Center of China in 2020. The maps were created using ArcGIS 10.2 (http://www.esri.com/software/arcgis/arcgis-for-desktop).

The Jing-Jin-Ji region located at 36.02° N–42.62° N latitude and 113.06° W–119.88° W longitude (Fig. 1), and covers nearly 2% of China’s total territory. The total population reached 112 million and produced nearly 10% of China’s Gross Domestic Product (GDP)^37^. With the rapid development of economy and population, the Jing-Jin-Ji region has become one of the metropolitan regions with serious water scarcity, conflict between rapid economic development and sustainability^38^. To reduce above issues, the Programme on the Beijing-Tianjin-Hebei Coordinated Development has been approved in April 2015, providing one innovative solution for regional coordinated development. The hydrological and meteorological conditions varied for different regions and periods because of the multifarious physiographic characteristics^37^. There is a tremendous conflict between water supply and demands because the water resources are limited but the water demands had increased dramatically. The annual average temperature is about 12 °C at southern part to nearly 2 °C at northern part of Jing-Jin-Ji region. The average annual precipitation is about 540 mm for past several decades, and nearly 70% of the annual precipitation concentrates in the flood season from June to September^35^. With the combined effects of climate variables, the water security encounters enormous pressure especially in Hebei province, and therefore affects the vegetation growth and agricultural production within the Jing-Jin-Ji region.

### Date source

The satellite-based VHIs used to assess the vegetation growth activity and drought conditions were derived from the National Oceanic and Atmospheric Administration (NOAA) Satellite and Information Service Global Area Coverage (GAC) dataset, which were produced based on the Advance Very High Resolution Radiometer (AVHRR) daily reflectance and emission in the visible, near infrared and two infrared bands including IR4 and IR5. The AVHRR was flown in NOAA polar-orbiting satellites including NOAA-7 (launched on June 23, 1981), NOAA-9 (December 12, 1984), NOAA-11 (September 24, 1988), NOAA-14 (December 30, 1994), NOAA-16 (September 21, 2000), NOAA-18 (May 20, 2005) and NOAA-19 (June 2, 2009). The 7-day composite temporal resolution and 16 km spatial resolution VHIs data were aggregated from original daily data for the period of 1982–2016. The weekly VHIs were processed to remove high frequency noise, which should be more suitable for climatology analysis. The 16 km spatial resolution of VHIs were used since the time series were longer^22^. The VHIs were extracted from blended vegetation health product which was reprocessed from AVHRR data (1981–2012) and blended Visible Infrared Imaging Radiometer Suite (VIIRS) data (2013–2016), which can be obtained from the Center for Satellite Applications and Research of NOAA at http://www.star.nesdis.noaa.gov/.

The Niño 3.4 indicating the regional average equatorial SST over the central tropical Pacific located at 5° S–5° N, 170° W–120° W^39^, was used as a substitution of ENSO to reveal the vegetation sensitivity in Jing-Jin-Ji region. It can be downloaded from National Weather Service Climate Prediction Center (CPC) of NOAA available at https://www.cpc.ncep.noaa.gov/data/indices/.

### Definition of satellite-based VHIs

Five categories of VHIs including smoothed NDVI (SMN, eliminated noise with respect to NDVI), smoothed Brightness Temperature (SMT, eliminated noise with respect to BT), VCI, TCI and VHI were used as substitutions to investigate the change patterns of vegetation growth activity, to detect the change characteristics of drought and to reveal the impacts of Niño 3.4 on the vegetation growth activity. Definitions of VHIs are shown in Table 1. NDVI indicates the property of green vegetation to emit and reflect solar radiation, defined as the reflectance difference between visible (VIS, Ch1, 0.58–0.68 μm) and near infrared (NIR, Ch2, 0.72–1.1 μm) bands of solar spectrum^40^. It is one of the most popular vegetation indices for monitoring short and long term variations of terrestrial vegetation productivity and land cover because it reflects leaf area index (LAI) and vegetation biomass. Positive values of NDVI indicate vegetated conditions and near zero values indicate non-vegetated conditions. Bigger value of NDVI indicates that large difference exists between VIS and NIR band, which substantially indicates greener and denser vegetation. The BT was converted from the IR4 defined as the infrared reflectance at band of 10.3–11.3 μm. The smoothed Normalized Difference Vegetation Index (SMN) and smoothed Brightness Temperature (SMT) are derived from no noise NDVI and no noise BT because the NDVI and BT could be affected by noises resulted from atmosphere constituents, unusual events, pre- and post-launch calibrations. Since different sources of noise affect NDVI and BT, it is hard to propose individual procedure for each source of noise. In general, the noise in NDVI and BT were removed empirically by comparing satellite and in situ observations using methods of mathematical statistics and validation, such as, Empirical Distribution Function (EDF) and statistical methods. More details of noise removal can refer to Kogan et al.^41^. After smoothing, the SMN can be used to assess the start and senescence of vegetation, detect the growing season, and at the same time, the SMT can be used to estimate thermal condition and cumulative degree days for vegetation growth. The interannual differences in SMN and SMT fluctuated due to the weather variation after smoothing, which were estimated relative to the maximum and minimum intervals of both NDVI and BT variations.

**Table 1:** Definition of VHIs.

VHIs	Full name	Definition	Range
NDVI	Normalized Difference Vegetation Index	NDVI = (NIR-VIS)/(NIR + VIS)	[− 1, 1]
BT	Brightness Temperature	IR4 for AVHRR, or IR5 for VIIRS	N/A
SMN	Smoothed NDVI	No noise NDVI	[−1, 1]
SMT	Smoothed BT	No noise BT	N/A
VCI	Vegetation Condition Index	VCI = 100 × (SMN-SMN_min_)/(SMN_max_ − SMN_min_)	[0, 100]
TCI	Temperature Condition Index	TCI = 100 × (SMT_max_ − SMT)/(SMT_max_ − SMT_min_)	[0, 100]
VHI	Vegetation Health Index	VHI = a × VCI + (1 − a) × TCI	[0, 100]

The VCI, TCI and VHI are used to assess the cumulative moisture, temperature and vegetation health conditions, respectively. The VCI is a proxy for moisture condition, based on the pre- and post-launch calibrated radiance converted to the SMN, which can be calculated as SMN anomaly relative to its multi-year climatology estimated based on bio-physical and ecosystem laws. The TCI is a proxy for thermal condition. It is calculated based on 10.3–11.3 µm AVHRR's radiance measurements converted to BT of land surface, which can estimate the hotness of the vegetation canopy. It is expressed as SMT anomaly with respect to multi-year climatology. Calculated based on the BT with completely removed high frequency noise, the value of TCI varies for hot or cold years. For example, in hot years with high temperature and insufficient water supply, the vegetation canopy can be overheating, which intensifies negative moisture deficit impacts on vegetation. The VHI indicates the combination of VCI and TCI to assess total vegetation health^5,42^. As shown in Table 1, the algorithms of VCI, TCI and VHI consist of comprehensive processing of smoothed NDVI and BT after removal of high frequency noise, enhancing seasonal period and calculation of climatology. The SMN_max_ and SMN_min_ (SMT_max_ and SMT_min_) are multi-year absolute maximum and minimum values of SMN (SMT), respectively. Coefficient *a* is used to quantify contribution of VCI and TCI to total vegetation health. It was assumed that the share of VCI and TCI into the VHI is equal and *a* = 0.5, because the share is uncertain for a specific location and time.

### Drought estimation using satellite-based VHIs

The satellite-based VHIs products can be used as proxy to monitor moisture condition, thermal conditions, vegetation health condition and drought. The duration, affected area and intensity of drought can be estimated based on different ranges of VHIs. The values of VHIs including VCI, TCI and VHI vary from 0 (extreme stress) to 100 (most favorable condition), with normal conditions during 40–60 corresponding to the average cumulative moisture, temperature and vegetation health conditions^25^. Higher values indicate better moisture, thermal or vegetation condition^22^. E.g., VCI < 40 indicates moisture stress, 40 < VCI < 60 for normal moisture condition, and VCI > 60 indicates favorable moisture condition. Decreasing trends of VHIs from 40 to 0 indicate intensification of vegetation stress, and vice versa for VHIs from 60 to 100. Further, drought-related stress can be assessed based on VCI, TCI and VHI if their values are less than 40. Taking VHI as an example, the VHI < 5 indicates exceptional drought intensity (D4), VHI < 15 for extreme-to-exceptional (D3-D4) drought intensity, VHI < 25 for severe-to-exceptional (D2–D4) drought intensity, and VHI < 35 indicates moderate-to-exceptional (D1–D4) drought intensity (Table 3).

### Cross wavelet transform and wavelet coherence

To reveal the impacts of Niño 3.4 on the VHIs, the non-stationary relationships between Niño 3.4 and VHIs were investigated using wavelet analysis. Wavelet transforms including Continuous Wavelet Transform (CWT) and its discrete counterpart can expand time series into time and frequency space, to identify both the dominant modes of variability and localized intermittent periodicities. The Cross Wavelet Transform (XWT) consisting of two CWTs is used to detect regions in time and frequency space where both time series show high common power. The Wavelet Coherence (WTC) between two CWTs is used to identify regions with significant coherence even though two time series do not necessarily have high comer power, and show the significant level against red noise backgrounds^43^. Details of CWT can be found in the previous studies^37,44,45^.

The XWT used to analyze the covariance of two time series X and Y, is defined as follows^44^:

1 $$ W_{n}^{{XY}} (s) = W_{n}^{X} (s) \times W_{n}^{{Y*}} (s) $$

where n and s indicate the time and scale of wavelet transform, $W_{n}^{X} (s)$ and $W_{n}^{Y} (s)$ are the wavelet transforms of X and Y_,_ and $W_{n}^{Y* } (s)$ denotes complex conjugation of $W_{n}^{Y} (s)$.

The XWT is further defined as $\left| {W_{n}^{{XY}} (s)} \right|$. The phase angle of $W_{n}^{{XY}} (s)$ indicates the phase relationships between X and Y in time and frequency space, and statistical significance is estimated with respect to a red noise^45^.

The WTC measures the coherence in time and frequency space between two CWTs, which is defined as follows^46^:

2 $$ R_{n}^{2} (s) = \frac{{\left| {S\left\langle {s^{{ - 1}} W_{n}^{{XY}} (s)} \right\rangle } \right|^{2} }}{{S\left\langle {s^{{ - 1}} \left| {W_{n}^{X} (s)} \right|^{2} } \right\rangle S\left\langle {s^{{ - 1}} \left| {W_{n}^{Y} (s)} \right|^{2} } \right\rangle }} $$

where S is the smoothing operator, $\left\langle  \cdot  \right\rangle$ smooth the wavelet spectrum in different time and scale, $R_{n}^{2} (s) \in [0,1][0,1]$, and $W_{n}^{{XY}} (s) = W_{n}^{X} (s) \times W_{n}^{Y * } (s)$. The smoothing operator S of $W_{n}^{{XY}} (s)$ in numerator and wavelet transforms in denominator is defined as follows^45^:

3 $$ S(W) = S_{{scale}} (S_{{time}} (W_{n} (s))) $$

where S_scale_ indicates smoothing along the wavelet scale axis and S_time_ denotes smoothing in time^44^:

4 $$ S_{{scale}} (W)|_{s}  = (W(t,s)c_{1} e^{{ - (t^{2} /2s^{2} )}} )|_{s} $$

5 $$ S_{{time}} (W)|_{n}  = (W(t,s)c_{2} \Pi (0.6s)|_{n} $$

where C_1_, C_2_ are the normalized constants, $\Pi$ is the rectangle function, and coefficient 0.6 is the empirically decorrelation length for Morlet wavelet. The wavelet phase difference is given as follows:

6 $$ \phi _{n} (s) = \tan ^{{ - 1}} \left( {\Im \left\{ {\left\langle {s^{{ - 1}} W_{n}^{{XY}} (s)} \right\rangle } \right\}/\Re \left\{ {\left\langle {s^{{ - 1}} W_{n}^{{XY}} (s)} \right\rangle } \right\}} \right) $$

where $\Im \left\{  \cdot  \right\}$ and $\Re \left\{  \cdot  \right\}$ are the imaginary part and real part of wavelet spectra, respectively.

The Monte Carlo method is used to estimate the statistical significance level of the WTC, with 5% significance level against red noise representing as a thick contour. The relative phase relationship is described as arrows with in phase (anti phase) pointing right (left). The cone of influence (COI) indicates the region of the wavelet spectrum in which edge effects become important, and it also signifies the wavelet power for a discontinuity at the edge drops that the edge effects are negligible beyond this point. However, it is noteworthy that the edge effects on and outside the COI cannot be ignored because the wavelet is not completely localized over the study period^43,44^.

### Trend analysis

The linear fitting regression and Mann–Kendall non-parametric methods were used to analyze the trends of VHIs. The linear trends of VHIs were calculated as follows:$y_{t}  = a + bt + e_{t}$, where y indicates the percentage of area of regional average weekly VHIs which is affected at different ranges at year *t* for the period of 1982–2016. The regression coefficient *b* is the linear slope of VHIs per year, and *e*_*t*_ is the residuals.

The nonlinear trends of VHIs were analyzed using the Mann–Kendall non-parametric test at a 5% significance level. The trend magnitude is estimated using Thiel-Sen approach, also known as Sen’s slope, defined as the median of all possible combinations for the time series. Details of Mann–Kendall non-parametric test and Sen’s slope can be found in previous studies^47^. The Mann–Kendal test was used to analyze the trends of VHIs during growing season, and the Sen’s slope was used to investigate the trend magnitudes of regional average monthly VHIs for the period of 1982–2016 in Jing-Jin-Ji region.

## Results

### Variations of regional average VHIs and their relationships

**Figure 2:**
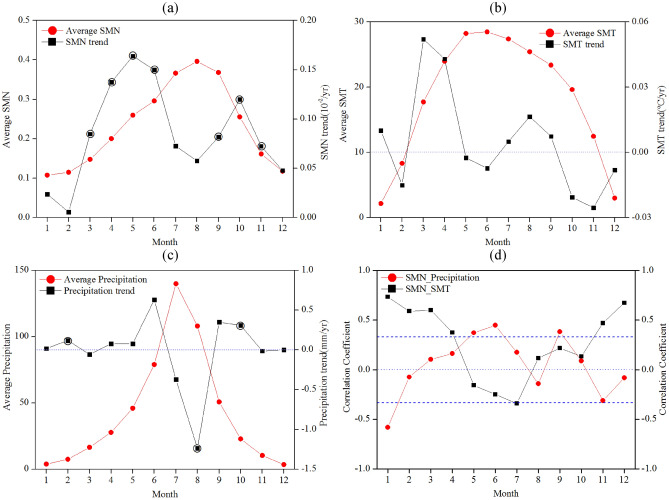
Thirty-five year regional average values and trend magnitudes of monthly SMN, SMT, precipitation and their correlations for the period of 1982–2016 in Jing-Jin-Ji region. (**a**) SMN; (**b**) SMT; (**c**) precipitation; and (**d**) Correlation coefficients between SMN and SMT/precipitation. The empty circles indicate statistically significant trends at 5% significance level, and the dash lines indicate that the SNM is significantly correlated with SMT or precipitation at 5% significance level.

Figure 2 shows the 35-year regional average values and trend magnitudes of monthly SMN (Fig. 2a), SMT (Fig. 2b), precipitation (Fig. 2c) and the Pearson's correlation coefficients between SMN and SMT (precipitation) (Fig. 2d) for the period of 1982–2016 in Jing-Jin-Ji region. The largest regional average value of monthly SMN occurred in August (0.3964), followed by September (0.3682) and July (0.3663). The regional average values of monthly SMN in winter season were smaller (from 0.1077 in January to 0.1177 in December) than other seasons for the period of 1982–2016, which mainly because that the vegetation growth is relatively weak due to the low temperature, especially, this region can be sometimes covered with snow in winter. The trend magnitudes of monthly SMN were positive throughout the year, indicating that the monthly SMN had increased for past 35-year period. Two peak values were found for trend magnitudes of monthly SMN, and the largest trend magnitude was detected in May (0.1640 × 10^–2^ /year), indicating that the regional average SMN in May had increased 0.0574. Two periods from March to June and from September to November had statistically significant increasing trends at 5% significance level. Similar with regional average values of monthly SMN, the trend magnitudes of SMN in winter season were smaller than those in other seasons. Surprisingly, the trend magnitude decreased from May to August, which might be caused by the shapely decreased precipitation (Fig. 2c) or other climate variables.

It is important to monitor the plant phenology using satellite-based SMN especially during growing season. Given that different SMN values at the beginning and end of the growing season are important for the satellite-based study of plant phenology. As used in previous studies^2,48^, this paper assumes 0.20 and 0.25 as the beginning and end of the growing season, respectively. It can be observed in Fig. 2a that the duration of growing season starts at approximate April and ends at about October, lasting about seven months. The vegetation growth peaked in the middle of the growing season (summer). The trend magnitudes of summer SMN were relatively smaller, but two peaks of trend magnitudes of SMN were found in the beginning and end of growing season, suggesting that the growing season had lengthened for past 35 years.

Regional average monthly SMT and precipitation showed similar patterns with SMN, larger values detected in summer, followed by spring and autumn, and relatively smaller values in winter. The largest value of SMT was detected in May (28.49 °C), followed by April (28.24 °C). However, the SMT during April and May had decreased for past 35-year period. The largest trend magnitude of regional average value of monthly SMT was detected in March with an annual rate of 0.05 °C, indicating that regional average value of SMT in March had increased up to 1.82 °C over past 35 years. The reasons for increasing trend of SMT might due to the global warming effects and urban heat island, since the urbanization and human activities for big cities such as Beijing city, Tianjin city and Shijiazhuang city have increased for past several decades. However, regional average values of SMT in several months had decreased, especially from October to December, which indicated that the warming trends had slowed down. No statistically significant trend was detected at 5% significance level (Fig. 2b). The largest value of monthly precipitation occurred in July (139.86 mm), followed by August (108.02 mm) and June (79.02 mm). However, the trend magnitudes of precipitation in July and August had decreased. The largest negative slope was detected in July with an annual rate of -0.37 mm, which indicated that regional average precipitation in July had decreased by 12.95 mm for the period of 1982–2016. Precipitation in three months including February, August and October had statistically significant trend at 5% significance level (Fig. 2c).

To further reveal the possible impacts of precipitation and temperature on SMN, the correlation coefficients between regional average SMN and SMT, SMN and precipitation were presented in Fig. 2d. For SMT, stronger positive correlation coefficients mainly occurred in winter and spring, with the largest correlation coefficient detected in January (0.7350), followed by December (0.6727). The significant correlations between SMN and SMT were detected from November to March at next year, and also in July at 5% significance level. The correlation coefficients between SMN and SMT in three months from May to July were negative, indicating that higher temperature might affect vegetation growth (Fig. 2b). On the other hand, the correlation coefficients between SMN and precipitation fluctuated from 1982 to 2016. The positive correlation coefficients mainly occurred in winter, during which time the vegetated areas might be affected by frost. The largest negative correlation coefficient was found in January (-0.5834). SMN in four months including January, May, June and September was significantly correlated to precipitation at 5% significance level. It is noteworthy that the SMN in August had also negatively correlated to precipitation, which might be caused by sharp decreasing trend of August precipitation (Fig. 2c).

It can infer that the SMN was mainly opposite correlated with SMT and precipitation, especially, in winter. E.g., the SMN is positively correlated with SMT and negatively correlated with precipitation from November in last year to February in the next year, indicating the dominate climate variable that affect the vegetation growth changed for different time period throughout the year. A comparative analysis of SMN trends (Fig. 2a) and correlation coefficients between SMN and SMT/precipitation (Fig. 2d) indicated that SMN variations were mainly dominated by SMT increase in March and April (Fig. 2b) and precipitation decrease in July and August (Fig. 2c). The effects of climate variables might delay by one or two months, because the largest trend magnitude of SMT occurred in March and May for SMN.

### Spatial and temporal variations of vegetation growth based on VHIs

**Figure 3:**
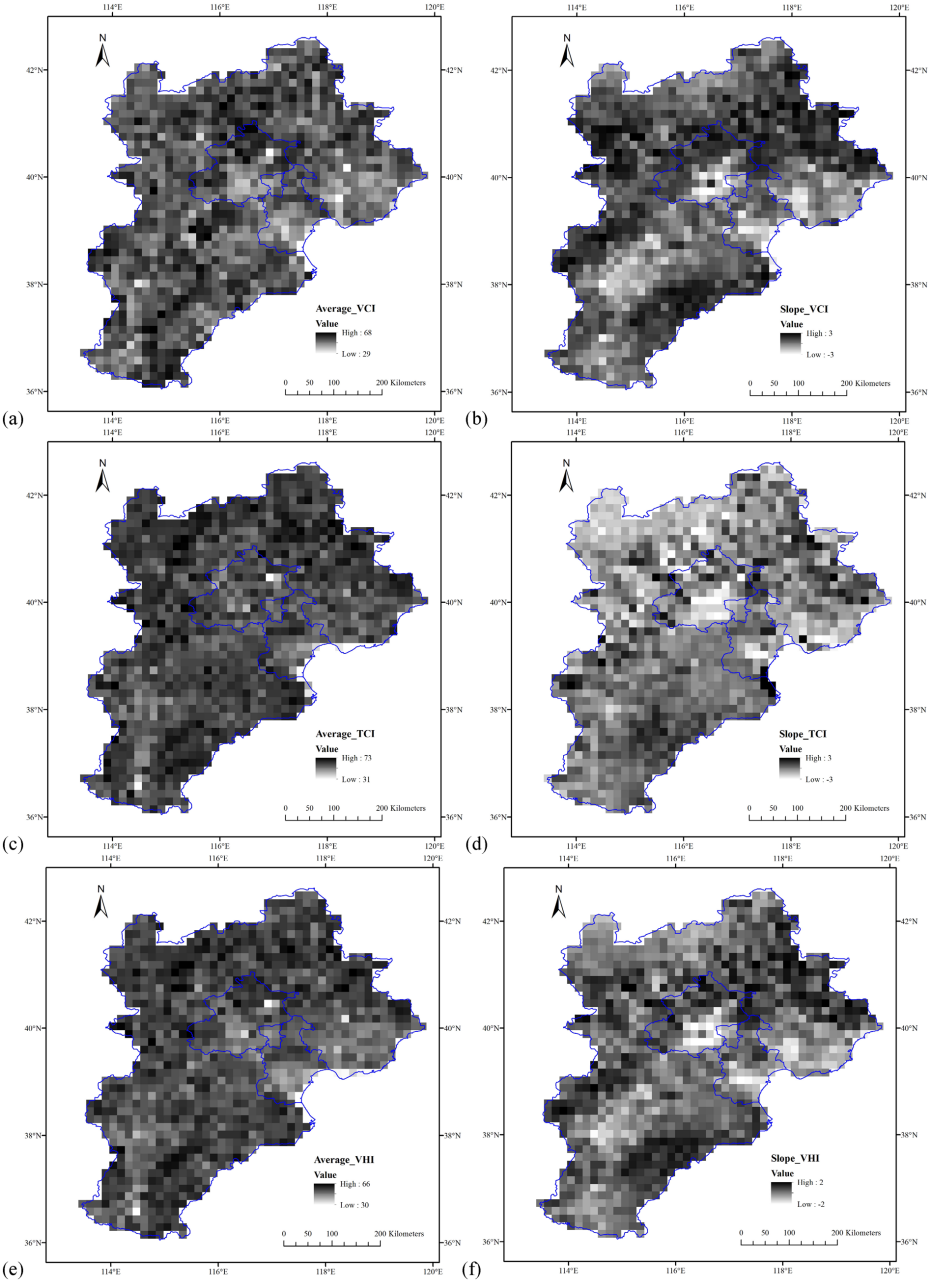
Spatial distributions of thirty-five year average values and slopes of VHIs during growing season for the period of 1982–2016 in Jing-Jin-Ji region. (**a**) average values of VCI, (**b**) trends of VCI, (**c**) average values of TCI, (**d**) trends of TCI, (**e**) average values of VHI, and (**f**) trends of VHI.

To reveal the spatial and temporal variations of vegetation growth activity in past decades, regional average values and Sen’s slopes of the VCI, TCI and VHI during growing season were studied for each individual grid between 1982 and 2016 in Jing-Jin-Ji region. As shown in Fig. 3, average values of VCI during growing season for each grid were between 29 and 68 (Fig. 3a), in comparison with the TCI within the ranges of 31–73 (Fig. 3b), and VHI from 30 to 66 (Fig. 3c), respectively. The slopes varied for different VHIs and different grids, with the maximum (2.4457) and minimum (-2.6147) values found in VCI (Fig. 3b), and the VHI had relatively small slopes (Fig. 3f). We further analyzed the significant test using Mann–Kendall non-parametric method, and the results show that the significant trends distributed scattered, which however, not the focus of this study.

Spatially, the results showed that about 77% of the Jing-Jin-Ji region had positive slopes for VCI (Fig. 3b), 38% based on the TCI (Fig. 3d) and 65% for VHI (Fig. 3f), indicating that the vegetation growth had enhanced in majority of Jing-Jin-Ji region for both VCI and VHI. However, the regions with increasing vegetation stress based on the VHI were wider than that of VCI. This was more evident in big cities such as Beijing city, Tianjin city and Shijiazhuang city (Fig. 3f), similar with that of VCI, indicating that those regions might sustain more moisture stress for vegetation growth. In case of TCI, the decreasing slopes mainly detected in the northern part, especially in Beijing city, Tianjin city and northwest of Jing-Jin-Ji region. Most of the southern part of the study area showed increasing trends, indicating that the southern part of Hebei province might be subjected to more thermal stress.

### Drought and vegetation growth condition estimation based on VHIs

**Figure 4:**
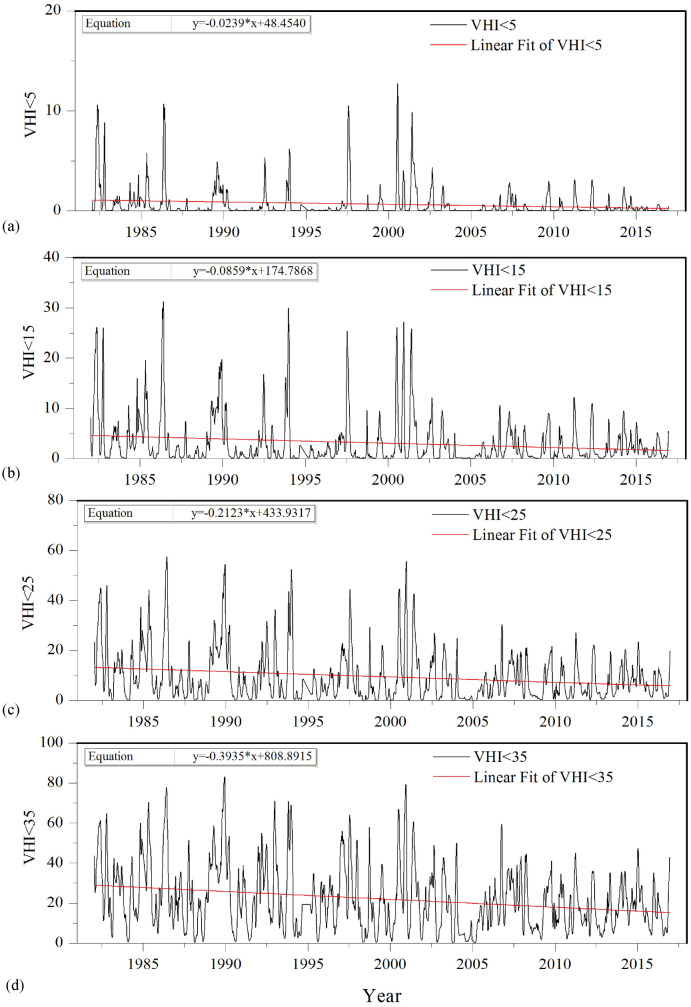
Percentage changes of area affected by drought at different intensities for the period of 1982–2016 in Jing-Jin-Ji region. (**a**) VHI < 5 indicates exceptional drought (D4); (**b**) VHI < 15 indicates extreme drought (D3); (**c**) VHI < 25 indicates severe drought (D2) and (**d**) VHI < 35 indicates moderate drought (D1).

It is very important for farmers and decision makers to know the drought intensity to estimate the potential disaster loss and to make drought relief measures, especially for severe drought. Regional moderate-to-exceptional (D1-D4) droughts were estimated based on regional average weekly VHI. Figure 4 shows percentage changes of area affected by drought at different intensities for the period of 1982–2016 in Jing-Jin-Ji region. Generally, the percentage of area that affected by drought had decreased from 1982 to 2016, and the slopes of linear fitting of percentage of area affected by drought had decreased from exceptional drought (-0.0239) to moderate drought (-0.3935). The average percentage of area affected by drought had decreased from moderate drought (22.03%) to exceptional drought (0.65%). It is worth mentioning that in some periods (e.g., during 2003–2005), no D3-D4 drought occurred, and the percentage of area affected by drought was relatively small after 2003. However, in some periods (e.g., during 2000–2001), droughts happened basically throughout the year. Especially, the percentage of area affected by exceptional drought (D4) reached the highest (12.75%) at nearly middle of 2000.

**Figure 5:**
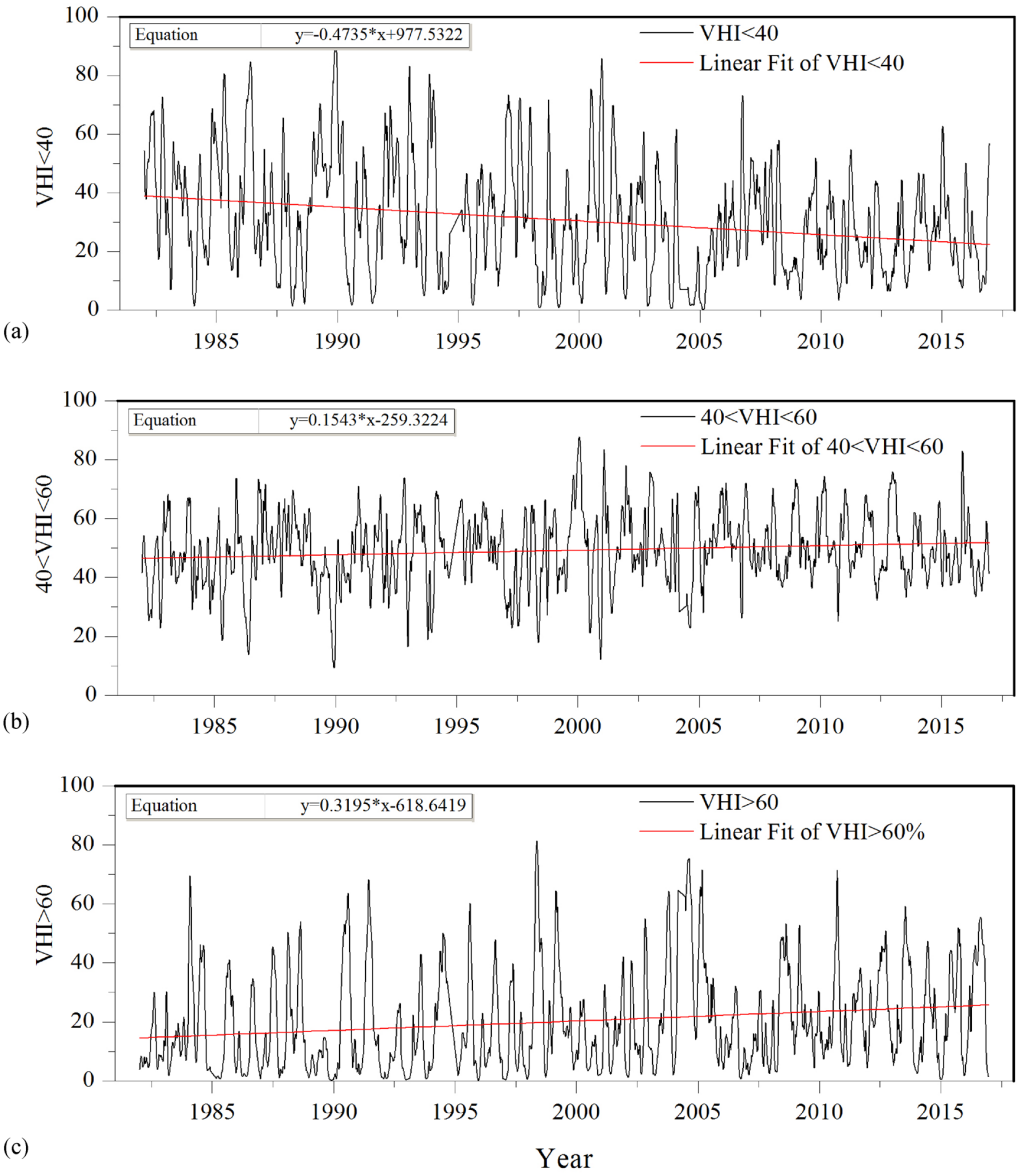
Percentage changes of area indicating vegetation stress, normal vegetation condition and favorable vegetation condition for the period of 1982–2016 in Jing-Jin-Ji region. (**a**) VHI < 40 indicates vegetation stress; (**b**) 40 < VHI < 60 indicates vegetation with normal condition; (**c**) VHI > 60 indicates vegetation with favorable condition.

The percentage changes of area indicating vegetation stress (VHI < 40), normal vegetation condition (40 < VHI < 60) and favorable vegetation condition (VHI > 60) were further studied, as shown in Fig. 5. Again, the percentage of area with vegetation stress (VHI < 40) had decreased from 1982 to 2016, and the slope of linear fitting (valued -0.4735) was larger than drought at other intensities (Fig. 5a). At the same time, the percentage of area with normal vegetation condition (Fig. 5b) and favorable vegetation condition (Fig. 5c) had increased from 1982 to 2016. To be more specific, the slope of linear fitting of percentage of area with favorable vegetation condition was bigger than that of normal vegetation condition, reached 0.3195 per year, indicating that the average value of percentage of area with favorable vegetation condition had increased up to nearly 11%, which might mainly because the vegetation coverage had increasing trends for past several decades, especially since the twenty-first century. Generally, the percentage of area fluctuated for VHI at different ranges. The largest percentage of area with favorable vegetation condition was detected in May of 1998 (79%), followed by August of 2004 (75%).

To reveal the effects of drought on the vegetation growth activity at different areas of Jing-Jin-Ji region, the percentage of area with regional average weekly VHI at different ranges in Beijing and Tianjin cities were also investigated for the period of 1982–2016. It is noteworthy that drought in some regions such as Beijing and Tianjin cities might affected by the urbanization. Based on highly resolution NDVI data derived from the Terra Moderate Resolution Imaging Spectroradiometer (MODIS) Vegetation Indices (MOD13Q1) Version 6 dataset, we have analyzed the change characteristics of NDVI for different land coverage. The results found that the urban area mainly increased in the 21^st^, especially after 2010. The urban area has increased from 6.92% in 2000 to 8.14% in 2010 and 12.11% in 2020. Thus, we should take into account the urbanization effects when analyze the drought condition. The results of Table 2 show that the average and maximum values of percentage of area affected by moderate-exceptional (D1-D4) droughts in Beijing city and Tianjin cities were slightly higher than those in Jing-Jin-Ji region, which might partly because of the urbanization effects, as we analyzed above. Further, the average and maximum values of percentage of area affected by D1-D4 droughts in Tianjin city were bigger than those in Beijing city. For example, the average (maximum) values of percentage of area affected by D4 drought were 28.72% (97.83%) in Tianjin city, 25.20% (91.46%) in Beijing city, and 22.03% (83.20%) in Jing-Jin-Ji region. The similar results can be detected for D2-D4 droughts. The linear fitting results provide the primary change of the percentage of area with regional average weekly VHI at different ranges, as shown in Figs. 4 and 5. The slope of linear fitting method indicated the trends for percentage of area affected by different ranges of VHI, with the positive value indicating increasing trend, and vice versa. The percentage of area had decreasing trends for all ranges of drought in Beijing city, which might because of the afforestation and forest planting activities. However, the slopes of linear fitting equation for D1-D4 droughts in Beijing city were bigger than those in Jing-Jin-Ji region, indicating that Beijing city suffered more severe drought stress than Jing-Jin-Ji region as a whole, which again might due to the relatively strong urbanization. At the same time, the slope of linear fitting for vegetation stress in Beijing city was bigger than that in Jing-Jin-Ji region. To the contrary, the slope of linear fitting for favorable vegetation condition was smaller than that in Jing-Jin-Ji region. However, the percentage of area with normal vegetation condition had decreased, differed from Jing-Jin-Ji region and Tianjin city. In case of Tianjin city, surprisingly, the percentage of area affected by D2–D4 drought intensity had increased, and the percentage of area with favorable vegetation condition (VHI > 60) had decreased from 1982 to 2016, indicating that the vegetation growth activity in Tianjin city became weaken, which might due to the rapid urban development for past several decades.

**Table 2:** Liner trends of percentage of area with regional average weekly VHI at different ranges for the period of 1982–2016 in Jing-Jin-Jin region, Beijing and Tianjin cities.

Region	VHI range	Linear equation	Trend	Average value (%)	Max value (%)
Jing-Jin-Ji region	VHI < 5	y = −0.0239x + 48.4540	Decrease	0.65	12.75
	VHI < 15	y = − 0.0859x + 174.7868	Decrease	3.07	31.26
	VHI < 25	y = − 0.2123x + 433.9317	Decrease	9.48	57.49
	VHI < 35	y = − 0.3935x + 808.8915	Decrease	22.03	83.20
	VHI < 40	y = − 0.4735x + 977.5322	Decrease	30.64	90.49
	40 < VHI < 60	y = 0.1543x − 259.3224	Increase	49.19	87.76
	VHI > 60	y = 0.3195x − 618.6419	Increase	20.17	81.38
Beijing City	VHI < 5	y = − 0.0055x + 11.6835	Decrease	0.72	22.80
	VHI < 15	y = − 0.0038x + 11.3928	Decrease	3.84	47.54
	VHI < 25	y = − 0.0215x + 54.5699	Decrease	11.55	72.05
	VHI < 35	y = − 0.1146x + 254.4341	Decrease	25.20	91.46
	VHI < 40	y = − 0.1793x + 392.5272	Decrease	34.04	96.02
	40 < VHI < 60	y = − 0.0163x + 79.5513	Decrease	46.95	94.85
	VHI > 60	y = 0.1956x − 372.1544	Increase	19.01	85.31
Tianjin City	VHI < 5	y = 0.0104x − 19.8853	Increase	0.80	28.83
	VHI < 15	y = 0.0324x − 60.8101	Increase	4.22	76.91
	VHI < 25	y = 0.0386x − 65.1976	Increase	12.95	94.13
	VHI < 35	y = − 0.0256x + 76.8262	Decrease	28.72	97.83
	VHI < 40	y = − 0.0535x + 145.6432	Decrease	38.63	98.85
	40 < VHI < 60	y = 0.0553x − 64.5055	Increase	46.07	82.28
	VHI > 60	y = − 0.0019x + 19.1741	Decrease	15.31	80.10

We further investigated change patterns of different vegetation conditions and corresponding duration for each grid in Jing-Jin-Ji region based on weekly VHI at different ranges including D1-D4, 0–40, 40–60 and 60–100, respectively. Generally, the spatial distributions of average values and their corresponding duration of VHI at each range were scattered and complex. The results of average values and their average corresponding duration of weekly VHI at each category during 1982–2016 are shown in Table 3. As expected, the average values and their corresponding average duration decreased from moderate drought (D1) to exceptional drought (D4), and increased from vegetation stress (VHI < 40) to normal vegetation condition (40 < VHI < 60) and finally favorable vegetation condition (VHI > 60). Taking moderate drought (D1) for an example, the average value of weekly VHI between 0 and 35 was 24.59 at an average duration of 398 weeks from 1982 to 2016 (nearly 80 days per year), in comparison with average VHI valued 28.24 at an average duration of 552 weeks from 1982 to 2016 (approximate 110 days per year). The results indicated an extensive distribution of vegetation stress for a long time (nearly 4 months) in Jing-Jin-Ji region. Compared with favorable vegetation condition (VHI > 60) with normal vegetation condition (40 < VHI < 60) and vegetation stress (VHI < 40), it can be detected that the average duration of weekly VHI between 40 and 60 was the longest (728 weeks), followed by 552 weeks for VHI < 40 and 492 weeks for VHI > 60. We can deduced that the vegetation growth activity was normal condition dominated with average weekly VHI value of 49.92 for the period of 1982–2016 in Jing-Jin-Ji region.

**Table 3:** Regional average value and their corresponding duration of weekly VHI at different ranges for the period of 1982–2016 in Jing-Jin-Ji region.

Category	VHI range	Average value	Duration(weeks)
D4	VHI < 5	2.01	14
D3-D4	VHI < 15	9.06	57
D2-D4	VHI < 25	16.88	174
D1-D4	VHI < 35	24.59	398
Stress	VHI < 40	28.24	552
Normal	40 < VHI < 60	49.92	728
Favorable	VHI > 60	71.33	492

**Figure 6:**
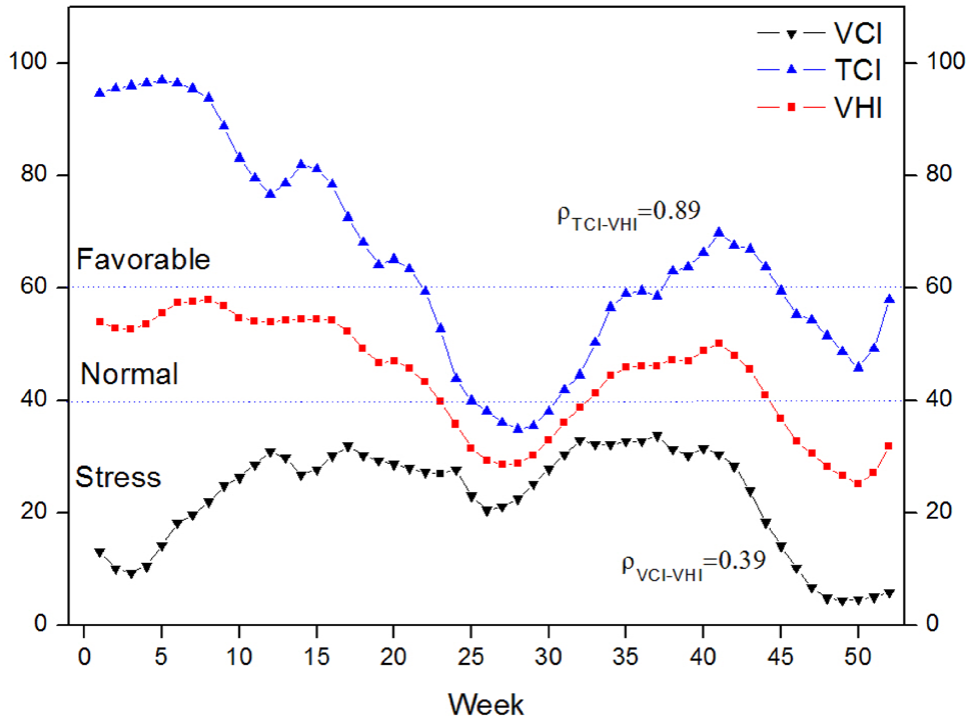
Dynamic change of regional average values of VHIs and their relationships in 2000 in Jing-Jing-Ji region.

Based on the relationships between drought and VHIs, the drought can be predicted 4–8 weeks in advance. Figure 6 illustrates the dynamic of regional average values of VHIs and their relationships in 2000 in Jing-Jin-Ji region for an example. The beginning of drought is identified in week of 23 when VHI crossed down 40 and end in week of 32, which lasted for 10 weeks. Based on the significantly positive correlation between TCI and VHI (ρ = 0.89), it might be possible to estimate the drought when TCI started to decrease, especially from fourteen week, which had several weeks before the drought happened. Considering the early detection of drought, it can provide early warning for farmers and decision maker and thus help to prepare precautionary measures for drought disaster in advance.

### Correlation analysis between Niño 3.4 and VHIs

**Figure 7:**
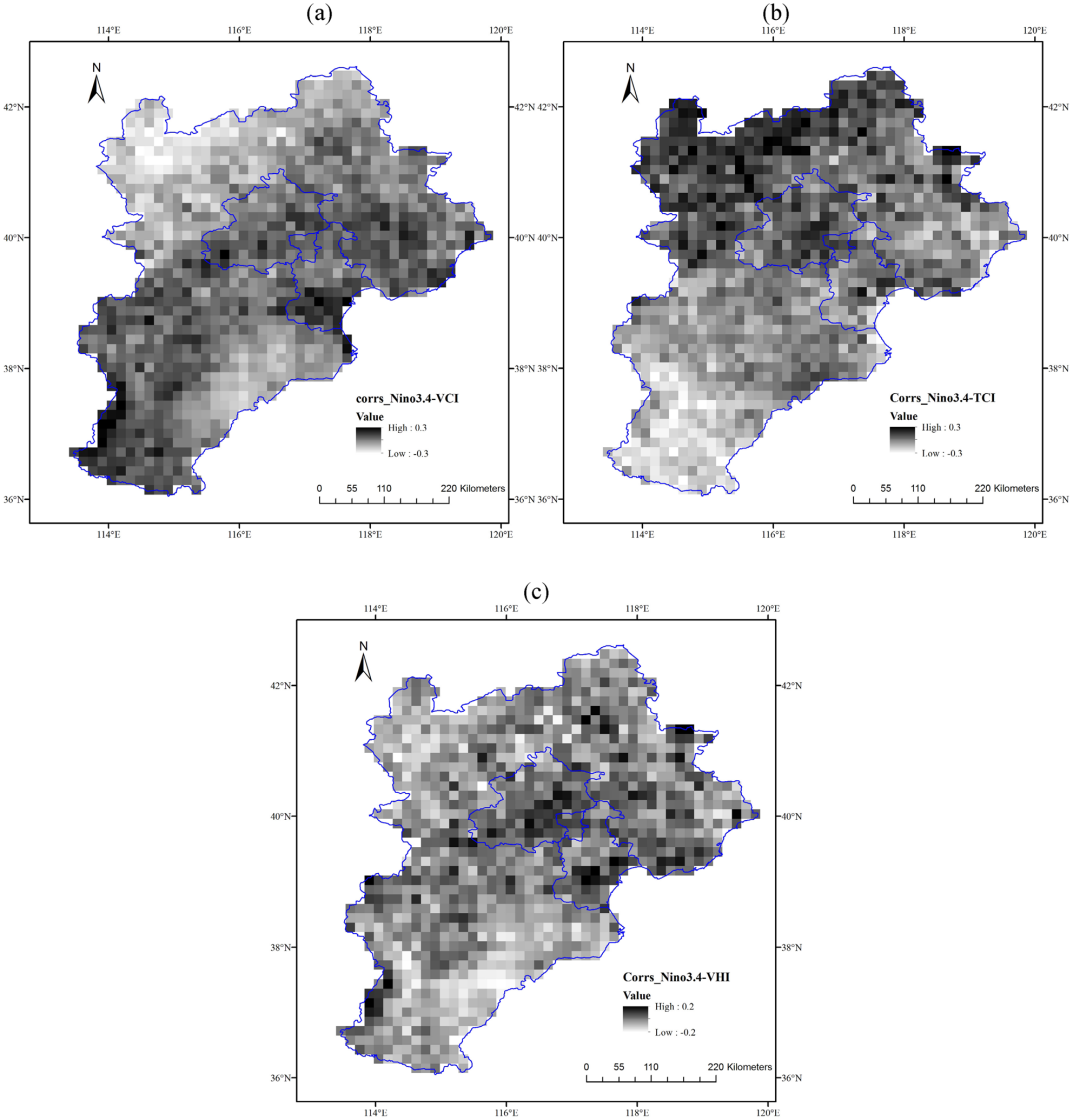
Correlation analysis between the Niño 3.4 and (**a**) VCI, (**b**) TCI, (**c**) VHI during growing season for the period of 1982–2016 in Jing-Jin-Ji region.

The Niño 3.4 was used as a substitution to reveal the sensitivity of regional vegetation health during growing season to ENSO by correlating the Niño 3.4 with VHIs for each grid during 1982–2016 in Jing-Jin-Ji region, as shown in Fig. 7. Generally, the relationships between Niño 3.4 and VCI were negatively dominated, with 65.25% of grids had negative correlation coefficients. The minimum value of correlation coefficient was -0.2638, located in the northwest of Jing-Jin-Ji region. Spatially, the negative correlations between Niño 3.4 and VCI were predominately located in the northwest and southeast of Jing-Jin-Ji region, and the grids irregularly located in the middle and southwest of Jing-Jin-Ji region were mainly positively correlated to Niño 3.4 (Fig. 7a).

On the contrary, for TCI, 67.71% of grids were positively correlated to Niño 3.4, with the maximum correlation coefficient ρ = 0.1997. The grids that exhibited positive correlation coefficients were mainly detected in Zhangjiakou and Chengde cities, located in the northern part of Jing-Jin-Ji region, especially in the northwest part. The negative correlation coefficients were mainly detected in the south of Jing-Jin-Ji region, such as Handan city and Xingtai city. The correlation coefficients were irregularly distributed in the middle of Jing-Jin-Ji region, which indicated that the Niño 3.4 exerted inconspicuous impacts on the thermal condition (Fig. 7b).

In case of VHI, the grids with positive (56.22%) and negative (43.78%) correlation coefficients were approximately equivalent. Spatially, the grids had positive and negative correlation coefficients between Niño 3.4 and VHI distributed more uniform than those of Niño 3.4 correlated to VCI and TCI. The grids located in the southeast and northwest of Jing-Jin-Ji region were negatively dominated, and the grids located in Beijing city, Tianjin city and some other sporadic areas had positive correlation coefficients (Fig. 7c). In comparison of Fig. 7a, b, it can be seen that the impacts of Niño 3.4 on VCI and TCI were opposite to some extent, especially in the south and northwest of Jing-Jin-Ji region, to partly counteract the impacts of Niño 3.4 on VHI (Fig. 7c).

### Nonlinear relationships between Niño 3.4 and VHIs using wavelet analysis

**Figure 8:**
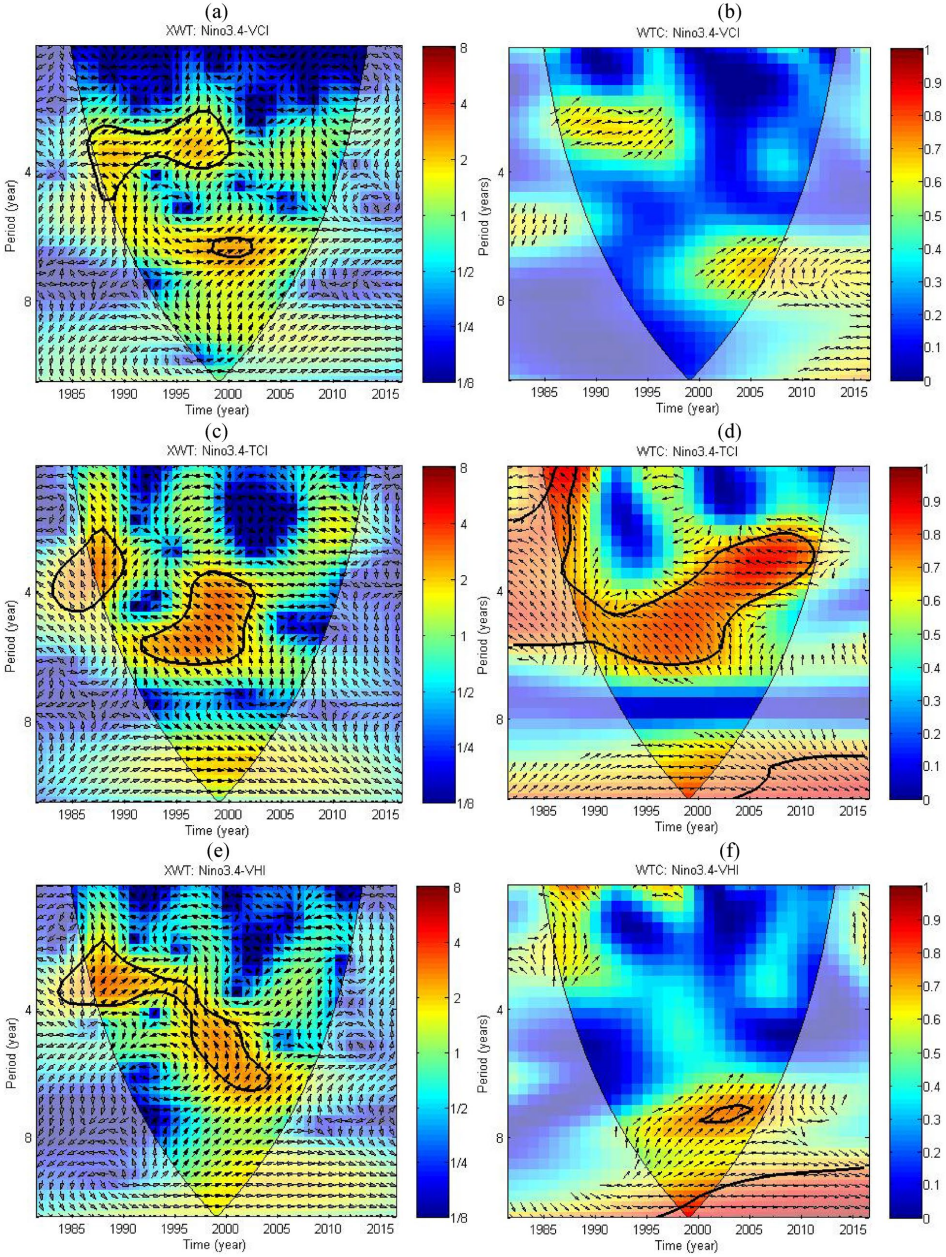
Cross wavelet transform and wavelet coherence between Niño 3.4 and regional average VCI (**a**,**b**), TCI (**c**,**d**), and VHI (**e**,**f**) during growing season for the period of 1982–2016 in Jing-Jin-Ji region. The sag dashed line draw through the wavelet spectrum is the cone of influence, and the thick enclosed areas indicate statistically significant wavelet coherence at a 5% significance level against a red noise. The arrows indicate the phase difference of period and coherence that are bigger than 0.5 between Niño 3.4 and regional average VCI. Left (right) pointing arrows indicate anti (in) phase coherence. The figures were created using Cross Wavelet and Wavelet Coherence Toolbox (http://grinsted.github.io/wavelet-coherence/).

Based on the linear relationships between Niño 3.4 and VHIs, the nonlinear relationships in time and frequency space between them were further studied using XWT and WTC. Figure 8 shows the XTW and WTC between Niño 3.4 and VCI, TCI and VHI. The 5% significance level against a red noise is shown using thick contour, and enclosed areas designate statistically significant coherence at 5% significance level with respect to a red noise background using Monte Carlo simulation. The relative phase relationship between two time series is described using arrows when phase difference exceeds 0.5. The vectors indicate the phase difference between Niño 3.4 and VHIs. A horizontal arrow point from left to tight indicates in phase, and an arrow points vertically upward signifies the second series lags the first by 90°, i.e., the phase angle is 270°, and vice versa when the arrows point left and downward.

Figure 8a shows the cross wavelet transform between Niño 3.4 and regional average VCI during growing season for the period of 1982–2016 in Jing-Jin-Ji region. The significant common power was detected mainly in the 2–6 year band between 1988 and 2000, also in the centered six year band during 2000s. The results show that Niño 3.4 and VCI are in phase for sectors with significant common power, and the phase relationship is in phase dominated outside the significant power areas. We can conclude that the Niño 3.4 is mainly positively correlated to VCI, which concurred with the results of correlation analysis as shown in Fig. 7a. Figure 8b shows scattered coherence between Niño 3.4 and VCI at mainly of 2–3 year band during 1990s and 6–8 year band after 2000, which is mainly located at the edge of COI and thus should be interpreted in cautious. It is noticeable that no significant common power was detected between Niño 3.4 and VCI for the period of 1982–2016, which might due to the relative weak correlation between them. However, the VCI was positively correlated to Niño 3.4 because the phase relationship is mainly in phase from Fig. 8b.

The vectors in Fig. 8c, d indicate the phase difference between Niño 3.4 and TCI at each time and period between 1982 and 2016. Large covariance between Niño 3.4 and TCI was detected at 2–6 year band, especially for wavelet coherence. The significant cross-wavelet common power was mainly detected during 1990s, which was anti phase dominated. Compared with wavelet coherence between Niño 3.4 and VCI (Fig. 8b), the coherence between Niño 3.4 and TCI were stronger at both inter-annual and inter-decadal oscillations. The significant coherence were mainly detected at 2–6 year band before 2010 and nearly 10 year band, which however located at the border or outside the COI, and therefore it might be subject to errors. Obviously, the significant wavelet coherence between Niño 3.4 and TCI was anti phase dominated within the COI based on their phase differences, which is also consistent with results of correlation analysis at Fig. 7b.

The cross wavelet transform between Niño 3.4 and VHI shows similar significant common power with respect to VCI and TCI, with nearly 2–6 year band before 2005, which mainly within the COI. However, the phase differences between them based on the common power are fickle, changing from about 45° in the 2–4 year band at the beginning of 1990s to nearly 135° in the 4–6 year band in 2000. The wavelet coherence between Niño 3.4 and VHI is relatively stronger than results of VCI but weaker than results of TCI at the same time, as shown in Fig. 8b, d. Two strong oscillations were detected including 1–4 year band before 1990 and 6–10 year band between 1990 and 2005 within the COI. However, the phase differences for above two oscillations are generally opposite, as we detected in Section "Correlation analysis between Niño 3.4 and VHIs" that the grids exhibited positive and negative correlations between Niño 3.4 and VHI were approximately equal (Fig. 7c). Significant wavelet coherence were also found before 2000, which located at the edge and outside of COI, again should be discarded.

Generally, relationships between Niño 3.4 and different VHIs including VCI, TCI and VHI are complex and unstable based on the results of correlation analysis in Section "Correlation analysis between Niño 3.4 and VHIs" and wavelet analysis in Section "Nonlinear relationships between Niño 3.4 and VHIs using wavelet analysis". The linear correlations between Niño 3.4 and VHIs varied for different grids and different VHIs, and the nonlinear wavelet common power and coherence likely change both in time and frequency. It seems that Niño 3.4 had more teleconnected to TCI than VCI, which might be interpreted that the Niño 3.4 exerted more direct impacts on the thermal than moisture condition.

## Discussion and conclusions

Using satellite-based VHIs, the paper assessed the spatiotemporal variability of vegetation growth activity for both regional average and individual grids, and estimated the change characteristics of drought for the period of 1982–2016 in the Jing-Jin-Ji region. To reveal the influence mechanism of vegetation growth activity, the linear and nonlinear relationships between VHIs and ENSO (using Niño 3.4 as a substitution) were further investigated using correlation and wavelet analysis. Our findings are summarized as follows:

### Assessment of vegetation growth based on VHIs

Generally, the vegetation growth activity has increased for past 35 years, especially after 2000. The monthly SMN had increased throughout the year especially during growing season. Analysis of related climate variables including SMT and precipitation showed similar change patterns with that of SMN. However, the trend magnitudes varied in different months for SMT and precipitation. The correlation analysis between SMN and SMT/precipitation indicated that the temperature is the dominant variable that affected the vegetation growth.

The spatiotemporal variations of VHIs for each individual grid showed that the VCI (TCI) was positive (negative) trends dominated during growing season, and the trend magnitudes varied for different VHIs and grids. The decreasing trends of VCI and VHI were mainly detected in big cities such as Beijing, Tianjin and Shijiazhuang, which should take into account the effects of urbanization since the urbanization level in the Jing-Jin-Ji region had increased a lot for past several decades, especially in large cities. The dominated positive trends of VCI and VHI revealed at Fig. 3 mainly concurred with results of Chen et al.^49^, who found a greening pattern especially prominent in China and India using satellite data for the period of 2000–2017. The VHIs were proposed by Kogan^42^ and had been widely used to assess the terrestrial vegetation productivity, land cover and drought characteristics. It is noteworthy that the VCI and TCI are normalization of smoothed NDVI and BT, which are the proxy for moisture and thermal conditions. The VHI is a proxy characterizing vegetation health, and can be calculated based on a combine estimation of moisture and thermal conditions. However, it still needs to further investigate whether it is reasonable to include both NDVI and BT in the vegetation health index. For example, the Land Surface Temperature (LST) is related to the albedo of the soil and vegetation, an important parameter to monitor the state of crops and vegetation, and also an important indicator of greenhouse effect at both global and local scales. It might be more suitable to indicate the thermal of the vegetation condition with respect to brightness temperature, especially for urban areas^50^, which should be useful to combine the LST into VHIs in the future. Another issue should note that the algorithms of VCI and TCI are based on comprehensive processing of smoothed NDVI and BT that remove the high frequency noise, and the algorithm of VHI is based on the weighted sum of VCI and TCI, which again need to interpret in cautious.

The strong relationships between SMN and SMT/precipitation provided the possibility to predict SMN using climate variables such as temperature and precipitation as predictors. The results agree with previous study of Jiang et al.^2^, who developed an artificial neural network calibrated by the genetic algorithm (ANN-GA) to estimate Alberta’s vegetation productivity using precipitation and temperature as predictors. However, the influence mechanism of vegetation growth was complex, which needs to be further investigated.

### Estimation of drought conditions based on VHIs

The relationships between VHI and drought intensity make it possibility of using VHI to monitor the start, duration and end of drought. As studied by Kogan et al.^5^, who used vegetation health indices for early detection and monitoring of drought. The VHIs have been successfully used for early drought detection and crop production losses estimation in 20 countries around the world. The intensity, duration, affected area of drought were estimated based on weekly VHI at different categories for both regional average values and each individual grid. In general, the percentage of area affected by drought at different intensities (D1-D4) had decreased, and the percentage of area with normal vegetation condition and favorable vegetation condition had increased from 1982 to 2016. However, the slopes of linear fitting of percentage of area affected by drought varied for different drought intensities, different regions and different time periods. It is noteworthy that the percentage of area affected by D2-D4 drought intensities and percentage of area with favorable vegetation condition had decreased from 1982 to 2016 in Tianjin city, indicating that the vegetation growth activity in some regions had become weaken, which might due to the urbanization effects and should be emphasized. The spatial distributions of average values and their corresponding duration of weekly VHI at each categories were mainly scattered and complex for each grid in Jing-Jin-Ji region.

### Relationships between Niño 3.4 and VHIs

The Niño 3.4 was mainly negatively (65.25% of grids) correlated to VCI, which were predominated located in the northwest and southeast of Jing-Jin-Ji region. However, the linear relationship between Niño 3.4 and TCI was positively dominated and 67.71% of grid had positive correlation coefficients mainly located in the northern part of Jing-Jin-Ji region. However, the spatial distribution of positive and negative correlation coefficients between Niño 3.4 and VHI was inconspicuous with respect to VCI and TCI, which might be caused by predominantly opposite effects of Niño 3.4 to VCI and TCI. The results are mainly concurred with previous studies. Kogan et al.^23^ evaluated the sensitivities of different land ecosystems by correlating VHIs with Niño 3.4 globally. The results indicated that satellite-based VHIs provided a combined contribution of moisture and thermal conditions with respect to cumulative vegetation response, which delineated more precisely the affected areas and period compared to weather-based features such as precipitation and temperature.

The nonlinear relationships between Niño 3.4 and three VHIs revealed using cross wavelet transform and wavelet coherence were in accordance with linear relationships revealed using correlation analysis. The nonlinear wavelet common power and coherences were unstable and complex, changing both in time and frequencies. It is expected that Niño 3.4 extracts more influences on temperature than precipitation, given the fact that ENSO is associated with variations in SST in the tropical Pacific Ocean^1^. The relatively weak relationships between Niño 3.4 and VHI might because Niño 3.4 had mainly positively affected the VCI with respect to mainly negative impacts on the TCI. However, the results varied at different regions and period, and the influence mechanism of the impacts of Niño 3.4 on the VHI still need to be further investigated.

### Future research

Our results in this study provide assessment of regional vegetation growth activity and drought estimation, which should be useful for the vegetation productivity assessment, drought detection and prevention, and also help to agriculture management for farmers and decision makers. Especially, the relationships between large-scale global anomalies and vegetation growth activity were further revealed using correlation and wavelet analysis, which expands our knowledge to predict vegetation and drought conditions using climate variables (e.g., precipitation and temperature) and large-scale global climate anomalies (e.g., Niño 3.4) as potential predictors. However, this study has limitations which need to be further investigated. For example, the trends of SMT, SMT and precipitation, the percentage changes of area affected by drought and different vegetation conditions were applied to regional average values. It is gratifying that this does not affect our mainly conclusions revealed above. As an extension of this study, our future work will therefore focus on the vegetation growth and drought assessment for different vegetation types and different climate division, and hopefully, a linear and nonlinear combined predicted model, as developed by in previous study of Jiang et al.^2^, might be applied to predict vegetation growth and drought conditions based on revealed VHIs-climate relationships in Jing-Jin-Ji region.
